# Utilization of cytologic cell blocks for targeted sequencing of solid tumors

**DOI:** 10.1002/cam4.5261

**Published:** 2022-09-20

**Authors:** Erica Vormittag‐Nocito, Ravindra Kumar, Kunwar Digvijay Narayan, Zhengjia Chen, Odile David, Frederick Behm, Gayatry Mohapatra

**Affiliations:** ^1^ Department of Pathology University of Illinois at Chicago Chicago Illinois USA; ^2^ Department of Pathology, Laboratory of Genomic Medicine University of Illinois at Chicago Chicago Illinois USA; ^3^ Department of Biostatistics University of Illinois at Chicago Chicago Illinois USA

**Keywords:** cell block, cytology, next‐generation sequencing, solid tumor malignancies

## Abstract

**Background:**

Targeted sequencing of cytologic samples has significantly increased in recent years. With increasing numbers of clinical trials for variant specific therapeutics, validating a comprehensive assay for cytologic samples has become clinically important.

**Aim:**

For this study, a retrospective review of cytologic cell blocks from fine needle aspirations and fluid specimens was performed.

**Methods:**

Two hundred twenty six total cases of solid tumor malignancies were identified, of which 120 cases and 20 lymph node negative controls were sequenced for the Oncomine Comprehensive Assay. Cytology and surgical specimen correlation was performed in a subset of cases. Statistical analysis to determine variant concordance was performed.

**Results:**

Within the 117 cases sequenced, a total of 347 pathogenic variants were detected. Of the 117 cases, 32 cases (27.4%) would qualify for FDA approved targeted therapy according to the current guidelines, and an additional 23 cases (19.7%) would qualify for clinical trial based on pathogenic variants detected.

**Discussion:**

With over 27% of cases in our cohort qualifying for some form of targeted therapy, our study shows the importance of providing comprehensive molecular diagnostic options. Despite only half of the cytology cases in the review period having enough material to be sequenced, overall approximately 27% of patients in this cohort would have benefitted from this service.

## Graphical abstract

This is a study of the application of cytologic cell block samples for comprehensive next generation sequencing. It demonstrates the usefulness of this sample type in the clinical setting and the importance of utilizing these samples for improved clinical outcomes.

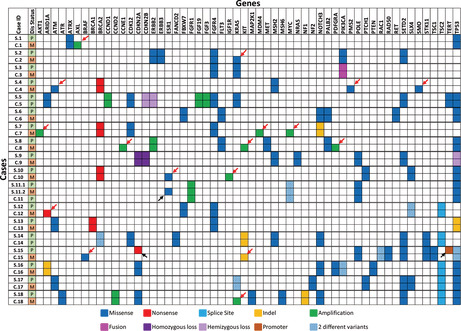

## 1 INTRODUCTION

Utilization of cytologic samples for clinical testing in the molecular pathology laboratory has greatly increased over recent years. As next‐generation sequencing (NGS) technologies have become dominant in the diagnostic and therapeutic arenas, it is essential to optimize assays for which low input of DNA can be used to sequence large numbers of clinically actionable targets in a highly accurate and reproducible manner. This will permit comprehensive overviews of the driver mutations for targeted therapy even in very small samples of tumor. Several publications have addressed the use of cytologic smear preparations and the minimum amount of tissue needed for adequate DNA/RNA extraction from a cytology sample.^1^, ^2^, ^3^ More recent studies have shown the potential of formalin‐fixed and paraffin‐embedded (FFPE) cytology cell blocks as an additional source of molecular testing material without the destruction of diagnostic cytologic smears.^4^, ^5^ In addition, there are preanalytic variables that are specific to cytology samples.^1^ With a small amount of tissue as seen in cytologic samples, variables such as tumor heterogeneity and low percentage of tumor cells may lead to false‐negative results. However, in cases of metastasis at the time of presentation or in cases where a fine needle aspiration (FNA) is the only procedure the patient can tolerate for sample collection, utilization of these small samples is invaluable for patient care.

For utilizing small samples, targeted panels are an ideal way to assess a tumor for clinically actionable mutations compared to PCR and Sanger sequencing methods. With many FDA‐approved drugs available for various types of solid tumors harboring mutations in *EGFR*, *BRAF*, *BRCA*, *NTRK*, *PIK3CA*, etc., sequencing these targets has never been more important for patient care. At our institution, a medium‐sized university hospital, we have elected to use a comprehensive solid tumor panel that targets important driver genes for therapy, tumor progression, and prognosis across all types of solid tumors. To our knowledge, this is the first study utilizing cytology cell blocks across multiple cancer types for comprehensive targeted sequencing for solid tumors to simulate routine diagnostic molecular oncology workflow. In the present study, we reviewed cytology cell blocks from 2013–2017 to identify cases with remaining malignant cells to be used for targeted panel sequencing. In order to test the viability of cytologic cell block samples as an alternative to surgical pathology material, archival cytology cell blocks from cytology fluid specimens and FNA material was utilized to simulate samples in a standard practice workflow.

## 2 MATERIALS AND METHODS

### 2.1 Case selection

Institutional Review Board approval for this study was obtained from the University of Illinois at Chicago. A case retrieval search was performed at the University of Illinois at Chicago pathology archives for all FNAs and cytology fluid samples that had a corresponding cytology cell block from August 2013 through May 2017. FNA specimens and fluid samples were chosen as all of these cases reflexively have a cell block performed. All samples were initially fixed in CytoLyt and transferred in pellet form into formalin for a minimum of 6 h prior to being placed on the processor in the histology laboratory. A total of 2702 cases were identified during this time period. The diagnoses of these cases were reviewed to identify all cases that had a malignant solid tumor neoplasm (this included carcinomas and soft tissue tumors). A total of 451 cases were identified as being positive for a solid tumor malignancy, and the cell block H&E slides for these cases were reviewed to confirm presence of malignant cells. Approximately 30% of these cases were found to have no malignant cells within the cell block sections and were confirmed to have had the diagnosis of malignancy rendered on the cytospin or FNA smear preparations; these cases were excluded from the study. Another 20% of cases identified were excluded from the study due to cell blocks or slides missing from the archives.

**FIGURE 1:**
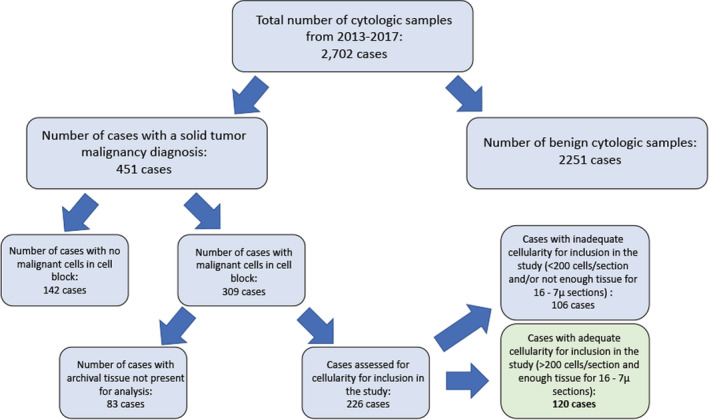
Sample selection schema

Of the remaining 226 cases, 106 (47%) cases were found to have inadequate numbers of tumor cells on cell block H&E sections (cases with less than 200 tumor cells per H&E section were considered inadequate and not included in the study) or inadequate tissue remaining on the paraffin block for 16 unstained slides to be cut for the molecular analysis performed in this study. A total of 120 cases were identified with enough archival tissue remaining to proceed with molecular analysis (Figure 1).

In addition to the cytology cell blocks for 120 cases, we reviewed the pathology archival material for any surgical pathology case for the patient cohort that had the same diagnosis as the cytology sample. Many of our cases within the cohort were cytologic fluid samples that represented metastatic disease where surgical resection was not an option (Table 1). In addition, our center is a tertiary care referral center and many smaller community practices send patients to our clinics for endoscopic diagnostic procedures, and therefore, resection specimens are not present in our archives for comparison in many lung and gastrointestinal malignancies. Due to these conditions, we were able to find surgical samples for only 18 cases that corresponded to the cytology sample and were included in the study for comparison of NGS results.

**TABLE 1:** Clinical characteristics of the samples included in the study

Case ID	Diagnosis	Age @ diagnosis	Gender	Tissue Type	Clinical/Pathologic Stage at Diagnosis	Clinical Hx (Hx)	Primary/ Metastasis
Case 1	Lung Adenocarcinoma	69	F	Pleural fluid, left	Stage IVB; pT4pM1c	Contralateral lung, bone, and brain metastasis at the time of diagnosis	Met
Case 2	Lung Adenocarcinoma	58	F	FNA, right hilar mass	pT2apN0	—	Recurrence
Case 3	Lung Adenocarcinoma	73	F	Pericardial fluid	Stage IVA; pT4pN3pM1a	Pleural fluid and pericardial fluid positive for malignancy at the time of diagnosis	Met
Case 4	Lung Adenocarcinoma	64	F	Pleural fluid	Stage IVB	Supraclavicular lymph node and brain metastasis at the time of diagnosis	Met
Case 5	Lung Adenocarcinoma	54	F	Pleural fluid	Stage IV	Spine metastasis at the time of diagnosis	Met
Case 6	Lung Adenocarcinoma	66	M	Pericardial fluid	Stage IIIA	Pericardial malignant effusion and positive lymph nodes at the time of diagnosis	Met
Case 7	Lung Adenocarcinoma	58	F	Pleural fluid	Stage IV	Bone metastasis at the time of diagnosis	Met
Case 8	Lung Adenocarcinoma	52	F	FNA, lymph node	at least Stage IIIB	—	Met
Case 9	Lung Adenocarcinoma	37	M	Pleural fluid	—	Malignant pleural effusion at the time of diagnosis	Met
Case 10	Lung Adenocarcinoma	80	F	Pleural fluid	—	—	Met
Case 11	Lung adenocarcinoma	37	F	Pericardial fluid	Stage IVB	—	Met
Case 12	Lung Adenocarcinoma	71	F	TBNA‐EBUS guided	Stage IV	—	Primary
Case 13	Squamous cell carcinoma	62	F	TBNA‐EBUS guided	Stage IIIB	—	Primary
Case 14	Lung Adenocarcinoma	60	F	TBNA‐EBUS guided	Stage IVA	Brain metastasis at the time of diagnosis	Primary
Case 15	Lung Adenocarcinoma	72	F	Pleural fluid	Stage IV	—	Met
Case 16	Lung Adenocarcinoma	73	M	TBNA‐EBUS guided, lung mass	—	—	Primary
Case 17	Lung Adenocarcinoma	55	F	TBNA‐EBUS guided, right bronchus intermedius mass	Stage IVB; pT2b pN3 pM1c	—	Primary
Case 18	Lung Adenocarcinoma	69	F	TBNA‐EBUS guided, right lower lobe lung mass	Stage IIIB, pT2b pN3	Original tumor diagnosed 7 years before presentation at an outside institution	Recurrence
Case 19	Lung Adenocarcinoma	61	F	TBNA‐EBUS guided	Stage IV	Metastasis known at the time of presentation	Primary
Case 20	Lung Adenocarcinoma	63	F	TBNA‐EBUS guided, mediastinal mass	Stage I, pT1 pN0	Original tumor 15 years prior to presentation	Recurrence
Case 21	Lung Adenocarcinoma	71	M	Pleural fluid	Stage IVB	Malignant pleural effusion at diagnosis	Met
Case 22	Lung Adenocarcinoma	65	M	Pleural fluid	at least Stage III	—	Met
Case 23	Lung adenocarcinoma	66	M	TBNA‐EBUS guided, left lower lobe lung mass	Stage IIIA	—	Primary
Case 24	Lung adenocarcinoma	68	M	TBNA‐EBUS guided, right hilar mass	Stage IV	Brain metastasis at the time of diagnosis	Primary
Case 25	Lung adenocarcinoma	50	M	Pleural fluid	Stage IVB	Malignant pleural effusion at the time of diagnosis	Recurrence
Case 26	Lung adenocarcinoma	75	M	TBNA‐EBUS guided, lymph node	Stage IV	—	Met
Case 27	Lung SqCC	61	M	TBNA‐EBUS guided, hilar mass	Stage IIIB	Hx of TB	Recurrence
Case 28	Lung SqCC	65	F	TBNA‐EBUS guided, lymph node	Stage IVA	Hx of LUL lung mass	Met
Case 29	Lung SqCC	64	M	TBNA‐EBUS guided, lymph node	At least Stage IIIB	Hx of right lower lobe lung mass	Met
Case 30	Lung SqCC	72	F	TBNA‐EBUS guided, mediastinal lymph node	Stage IIIB	Hx of smoking with lung mass	Met
Case 31	Lung SqCC	69	M	TBNA‐EBUS guided, right lung mass	Stage IV	—	Primary
Case 32	Lung SqCC	55	M	TBNA‐EBUS guided, lung mass	Stage IIIA	—	Primary
Case 33	Lung SqCC	62	M	TBNA‐EBUS guided, RUL mass	At least Stage IIIA	—	Primary
Case 34	Lung SqCC	82	F	TBNA‐EBUS guided, left lung mass	At least Stage III	—	Primary
Case 35	Small cell carcinoma, Lung	59	M	Pleural fluid	Stage IV	—	Met
Case 36	Small cell carcinoma, Lung	62	M	TBNA‐EBUS guided, lymph node	Stage IV	Mediastinal lymphadenopathy & spinal lesions at the time of presentation	Met
Case 37	Small cell carcinoma, Lung	57	F	Pericardial fluid	Stage IV	Brain metastasis at the time of diagnosis	Met
Case 38	Small cell carcinoma, Lung	63	M	TBNA‐EBUS guided, lymph node	Stage IV	Brain metastasis at the time of diagnosis	Met
Case 39	Small cell carcinoma, Lung	71	M	TBNA‐EBUS guided, lymph node	—	Confined to chest cavity at the time of diagnosis	Met
Case 40	Small cell carcinoma, Lung	76	F	TBNA‐EBUS guided, right hilar mass	T2N1	Confined to chest cavity at the time of diagnosis	Primary
Case 41	Poorly differentiated lung carcinoma	50	F	TBNA‐EBUS guided, lymph node	Stage IV	Adrenal, hilar lymph nodes, and brain metastasis at the time of diagnosis	Met
Case 42	Poorly differentiated lung carcinoma	54	F	TBNA‐EBUS guided, lymph node	Stage IV	Mediastinal lymph nodes, brain, and spinal cord metastasis at the time of diagnosis	Met
Case 43	Poorly differentiated lung cancer	64	M	TBNA‐EBUS guided, RUL lung mass	Stage IIIC	—	Primary
Case 44	Adenocarcinoma unknown origin	56	F	Pleural fluid	Stage IV	Brain and spinal cord metastasis at the time of diagnosis.	Met
Case 45	Adenocarcinoma unknown origin	59	F	Pleural fluid	Stage IV	Positive lymph nodes and pancreatic lesion at diagnosis; presumed primary was lung mass	Met
Case 46	Adenocarcinoma unknown origin	60	M	Ascitic fluid	—	Malignant ascites on presentation	Met
Case 47	Adenocarcinoma unknown origin	49	F	Ascitic fluid	Stage IV	Omentum lesions and malignant ascites on presentation	Met
Case 48	Adenocarcinoma unknown origin	49	M	Pleural fluid	Stage IV	Liver and bilateral lung lesions on presentation	Met
Case 49	Mucinous adenocarcinoma unknown primary	70	F	Pleural fluid	Stage IV	Spinal metastasis at presentation	Met
Case 50	High‐grade serous ovarian adenocarcinoma	46	F	Ascitic fluid	pT3cpN1b	—	Met
Case 51	High‐grade serous ovarian adenocarcinoma	86	F	Pleural fluid	Stage IVA	Pelvic lymph nodes positive with abdominal and peritoneal implants on presentation	Met
Case 52	High‐grade serous ovarian adenocarcinoma	65	F	EUS FNA, gastric mass	Stage IV	Vaginal wall invasion and stomach and spleen lesions at the time of diagnosis	Met
Case 53	High‐grade serous ovarian adenocarcinoma	56	F	Pleural fluid	Stage IV	Lung and Liver metastasis at the time of diagnosis	Met
Case 54	High‐grade serous ovarian adenocarcinoma	77	F	Ascitic fluid	Stage III	Omentum and abdominal wall lesions at the time of diagnosis	Met
Case 55	High‐grade serous ovarian adenocarcinoma	59	F	Pleural fluid	Stage IV	Omentum lesions at the time of diagnosis	Met
Case 56	High‐grade serous ovarian adenocarcinoma	68	F	Pleural fluid	Stage IIIC	Carcinomatosis at the time of diagnosis	Met
Case 57	High‐grade serous ovarian adenocarcinoma	60	F	Ascitic fluid	Stage IIIC	Carcinomatosis at the time of diagnosis	Met
Case 58	High‐grade serous ovarian adenocarcinoma	49	F	Pleural fluid	Stage IV	Abdominal wall implants at the time of diagnosis	Met
Case 59	Low‐grade serous ovarian carcinoma	41	F	Pelvic washing	Stage IIIB, pT3bpN1a	—	Met
Case 60	Endometrial adenocarcinoma	60	F	Ascitic fluid	Stage IV, pT3apN0pM1	Serous endometrial carcinoma	Met
Case 61	Endometrial adenocarcinoma	49	F	Ascitic fluid	Stage IV	Presented with carcinomatosis	Met
Case 62	Endometrial adenocarcinoma	78	F	Ascitic fluid	Stage IV	Clear cell endometrial carcinoma, presented with carcinomatosis	Met
Case 63	Endometrial adenocarcinoma	59	F	Ascitic fluid	Stage IV	Serous endometrial carcinoma	Met
Case 64	Endometrial adenocarcinoma	58	F	FNA, left lower back soft tissue mass	Stage IV	Serous endometrial carcinoma; back mass and small intestine mass at the time of diagnosis	Met
Case 65	Endometrial adenocarcinoma	53	F	Pelvic washing	Stage IV	Endometrioid adenocarcinoma with lung and diaphragm metastasis at the time of diagnosis	Met
Case 66	Endometrial adenocarcinoma	62	F	Ascitic fluid	Stage IVB, pT3pM1	Serous endometrial carcinoma with lung metastasis at presentation	Met
Case 67	Endometrial adenocarcinoma	64	F	Pleural fluid	Stage IV	Pleural effusion and lung metastasis at the time of presentation	Met
Case 68	Cervical SqCC	62	F	FNA, left supraclavicular mass	Unknown	Original tumor treated at outside hospital. Presented 10 years after original diagnosis with positive lymph node	Met
Case 69	Cervical SqCC	59	F	Ascitic fluid	Stage IV	Hx of recurrent low saag ascites, Hx of Cervical cancer	Met
Case 70	Cervical adenocarcinoma	37	F	Ascitic fluid	Stage IIB	History of treatment at outside hospital. Presented with omentum lesions and supraclavicular lymph node positive.	Met
Case 71	Breast adenocarcinoma	55	F	Pleural fluid	Stage IV	Bone, lung, brain, and gastric metastasis at the time of diagnosis	Met
Case 72	Breast adenocarcinoma	65	F	Pleural fluid	Unknown	Diagnosis at another institution more than 15 years before presentation. Presented to our institution with lung and liver metastasis	Met
Case 73	Bilateral breast adenocarcinoma	57	F	Pleural fluid	pT1c	Presented more than 10 years after initial diagnosis with spine and brain metastasis and pleural effusion	Met
Case 74	Breast adenocarcinoma	48	F	Pleural fluid	Stage IIB	Cytologic sample 4 years after original diagnosis of lung metastasis	Met
Case 75	Breast adenocarcinoma	56	F	FNA, left breast mass	at Least Stage III	Positive lymph nodes at the time of presentation	Primary
Case 76	Breast adenocarcinoma	62	F	Ultrasound‐guided FNA, liver	Unknown	Presented to our institution 12 years after initial diagnosis with spine metastasis	Met
Case 77	Breast adenocarcinoma	73	F	TBNA‐EBUS guided, RLL mass	Unknown	Hx of breast cancer with unknown time of diagnosis, presented with lung nodule	Met
Case 78	Pancreatic adenocarcinoma	91	M	EUS‐guided FNA, pancreatic body	Stage IV	Liver and lung metastasis at the time of diagnosis	Primary
Case 79	Pancreatic adenocarcinoma	68	F	EUS‐guided FNA, pancreas	Stage IA	—	Primary
Case 80	Pancreatic adenocarcinoma	63	M	EUS‐guided FNA, liver	Stage IV	Liver and lymph node metastasis at the time of diagnosis	Met
Case 81	Pancreatic adenocarcinoma	79	F	EUS‐guided FNA, liver	Stage IV	Liver lesions at the time of diagnosis	Met
Case 82	Pancreatic carcinoma with squamoid features	64	F	EUS‐guided FNA, pancreas	Stage IV	Liver lesions at the time of diagnosis	Primary
Case 83	Pancreatic adenocarcinoma	75	F	EUS‐guided FNA, pancreatic head	Stage IV	Liver and lung metastasis at the time of diagnosis	Primary
Case 84	Pancreatic adenocarcinoma	71	F	EUS‐guided FNA, pancreas	Unknown	Only seen for diagnostic procedure	Primary
Case 85	Pancreatic adenocarcinoma	59	M	EUS‐guided FNA, pancreatic head	T3	—	Primary
Case 86	Pancreatic adenocarcinoma	58	M	EUS‐guided FNA, pancreas	Unknown	—	Primary
Case 87	Gastrointestinal stromal tumor (GIST)	45	F	EUS‐guided FNA, stomach	pT2pN0	—	Primary
Case 88	Gastrointestinal stromal tumor (GIST)	75	M	EUS‐guided FNA, splenic hilum	Unknown	—	Primary
Case 89	Gastrointestinal stromal tumor (GIST)	61	F	EUS‐guided FNA, mediastinal mass	Stage IV	Liver metastasis at the time of diagnosis	Primary
Case 90	Gastrointestinal stromal tumor (GIST)	59	M	EUS‐guided FNA, gastric mass	Unknown	—	Primary
Case 91	Gastrointestinal stromal tumor (GIST)	67	M	EUS‐guided FNA, stomach	pT2	—	Primary
Case 92	Gastrointestinal stromal tumor (GIST)	48	M	EUS‐guided FNA, abdominal mass	Unknown	Patient diagnosed ten years earlier than the presentation. FNA performed on recurrence	Primary
Case 93	Extrahepatic cholangiocarcinoma	52	M	EUS‐guided FNA, pancreas	Stage IIIA	—	Primary
Case 94	Cholangiocarcinoma	82	F	Ascites fluid	Stage IV	Malignant ascites at the time of diagnosis	Met
Case 95	Gastric adenocarcinoma	67	F	Ascites fluid	Stage IV	Lymph nodes positive and malignant ascites at the time of diagnosis	Met
Case 96	Gastric adenocarcinoma	73	M	Pleural fluid	Stage IIIA, pT2pN3a	—	Met
Case 97	Low‐grade appendiceal mucinous tumor (LAMN)	52	M	Peritoneal fluid	pT4b pN2	Liver lesions and peritoneal implants at the time of presentation	Met
Case 98	Head & Neck SqCC	58	M	Pleural fluid	Unknown	Original tumor treated at outside institution. Presented with pleural effusion and lung nodule.	Met
Case 99	EBV‐associated nasopharyngeal carcinoma	56	M	FNA, left neck mass	pT2pN2b	Lymph Node positivity at time of diagnosis	Met
Case 100	Laryngeal SqCC	54	M	FNA, RUL mass	pT4apN0	Hx of laryngectomy for SqCC; Biopsy of lung mass found to be metastasis	Met
Case 101	Left frontal sinus SqCC	49	M	TBNA‐EBUS guided, lymph node	pT4apN0pM0	Hx of sinus carcinoma. FNA of lung metastasis	Met
Case 102	Tongue/tonsil/FOM SqCC	63	M	FNA, right neck mass	Stage IVB	—	Met
Case 103	SqCC of face (skin)	87	M	FNA, submandibular region, left	pT2pN0	Hx of Mycosis Fungoides	Met
Case 104	SqCC of larynx	64	F	FNA, right neck mass	Stage IVA; pT2pN2b	—	Met
Case 105	Conjunctival SqCC	78	F	FNA, right preauricular region	pT4pN1	Neck lymph node FNA	Met
Case 106	Metastatic papillary thyroid carcinoma	66	F	TBNA‐EBUS guided, posterior tracheal mass	Stage III, pT4 pN1a	Current presentation after treatment with radioactive iodine and resection of thyroid	Met
Case 107	Papillary thyroid Carcinoma	27	F	US‐guided FNA, left cervical lymph node	—	—	Met
Case 108	High‐grade salivary carcinoma	74	M	TBNA‐EBUS guided, lymph node	At least Stage III	Hx of salivary duct carcinoma; mediastinal lymph node positive	Met
Case 109	Melanoma	38	M	Ascites fluid	Unknown	Presented 5 year after original diagnosis with malignant ascites and spine lesions	Met
Case 110	Melanoma	43	M	Peritoneal fluid	Unknown	Presented 12 years after original diagnosis with metastatic bone lesions	Met
Case 111	Metastatic renal cell carcinoma	57	M	Right rib fluid	Unknown	Prior outside treatment of original tumor. Presented with bone metastasis	Met
Case 112	Oncocytic renal cell carcinoma	65	M	CT‐guided FNA, right kidney	Stage I, kidney confined lesion.	Hx of right lung cancer, radioablated renal mass; no resection performed	Primary
Case 113	Urothelial adenocarcinoma	64	M	CT‐guided FNA, Left acetabulum	pT3apN0 (Bladder tumor); Stage IV (DLBCL)	Hx of Diffuse large B cell lymphoma with prior treatment and BCG treatment of urothelial carcinoma before resection. This sample was at the time of recurrence in bone	Met
Case 114	Malignant mesothelioma	65	M	Pleural fluid	—	—	Met
Case 115	Pancreatic neuroendocrine tumor	71	M	EUS‐guided FNA, pancreas	—	—	Primary
Case 116	Neuroendocrine tumor, duodenum	66	F	EUS‐guided FNA, duodenal bulb	Stage IV; pT3pN1pM1	Liver metastasis at the time of diagnosis	Primary
Case 117	Neuroendocrine tumor	77	F	EUS‐guided FNA, ampulla	Stage IV; pM1	Spine and liver metastatic lesions at the time of diagnosis	Met

A total of 20 negative controls were identified in FNA lymph node samples; 10 negative lymph nodes from patients with no history of malignancy, and 10 negative lymph nodes from patients with a history of malignancy were included.

### 2.2 DNA and RNA extraction

FFPE cytology cell blocks were sectioned at 7 μm. A total of 15 unstained and unbaked serial sections were used for both DNA and RNA extraction. A 5 μm section was stained with hematoxylin and eosin. Tumor area was marked, and the percentage of tumor cells was estimated by a pathologist. Tumor cells were manually macrodissected to enrich for tumor fraction to achieve ≥20% tumor cells in the DNA/RNA sample. The Promega semi‐automated FFPE DNA and RNA extraction kits were used with the Promega Maxwell RSC system (Promega Corporation). Quantitation of DNA and RNA by Qubit (Thermo Fisher Scientific) was performed prior to library preparation.

### 2.3 Targeted panel sequencing

Library preparation and sequencing using the Oncomine Comprehensive Assay version 3 was performed on all cases, using 20 ng of DNA and 20 ng of RNA for each sample, as has been previously described.^6^ The assay targets 161 unique genes including 84 genes for hotspot mutations, 43 genes for focal copy number gains, 48 full coding sequences for deletion mutations, and 51 fusion drivers. Data analysis was performed using the Torrent Suite software version v5.10. Ion Reporter version v5.10 was used for variant calling. A minimum average depth of coverage of 600X was considered adequate for each sample. Fusion analysis was performed with Ion Reporter version v5.10 fusion analysis workflow. Variants were classified as benign, likely benign, variant of undetermined significance, likely pathogenic or pathogenic based on the clinical criteria set by the College of American Pathologists and the Association of Molecular Pathology.^7^

### 2.4 Statistical analysis of outcome data

For cytologic and surgical comparison, results from a total of 17 paired samples were included. For each subject, the locus, genes, AA change, and values for variants (surgical and cytology) were recorded. The goal of this analysis was to determine the correlation between variants detected in surgical and cytology samples. Pearson's and Spearman's correlations were measured between variants. Parametric paired *t*‐test was performed to compare the means of variants. Nonparametric paired test (Wilcoxon Signed Rank Test) was used to compare the medians of variants. The significance levels were set at 0.05 for all tests. The SAS 9.4 Version (SAS Institute, Inc.) was used for data management and analyses.

A clinical chart review was performed for all cases that passed quality metrics. A total of 117 subjects were included in this analysis. The charts were reviewed for the following information: date of diagnosis, final pathology diagnosis, treatment regimen, date of recurrence, date of metastasis diagnosis, date of death, and date of last known contact. Time of overall survival (OS) was calculated as the time from study enrollment to death or last contact. Time of progression‐free survival (PFS) was calculated as the time from study enrollment to disease progression date, death date, or last contact whichever comes first. The survivor functions for PFS or OS were estimated by Kaplan‐Meier survival analysis. Cox proportional hazards model was employed to estimate the adjusted effect of variants, diagnosis, and metastasis status on PFS or OS after adjustment for all other factors.

## 3 RESULTS

### 3.1 Case cohort

**FIGURE 2:**
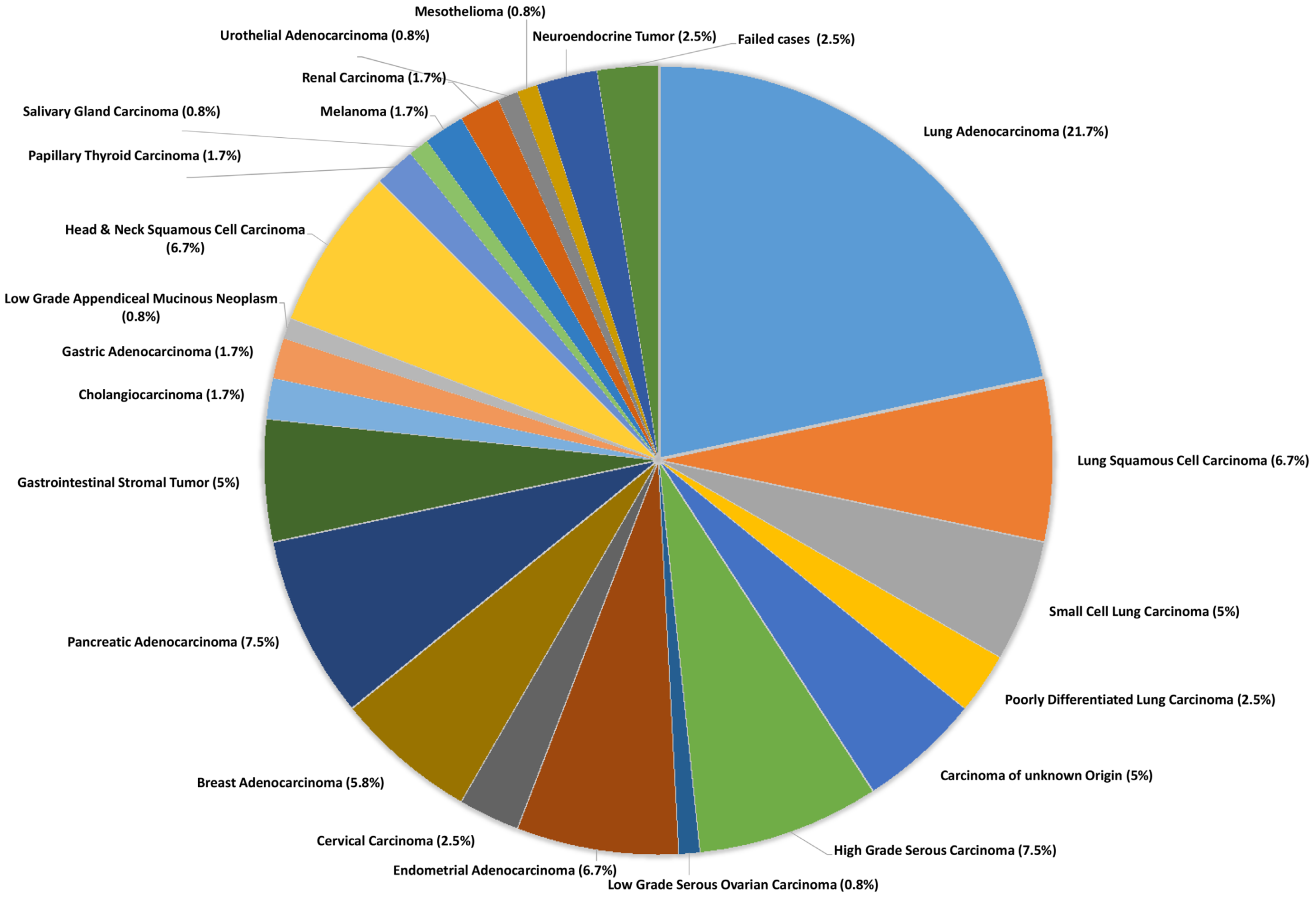
Cohort by diagnosis

A total of 120 cases met the criteria for inclusion in the study. Two samples did not yield enough DNA for library preparation, and one case had too much formalin‐induced artifacts for reliable analysis, leaving 117 samples in our study. There were 31 primaries, 6 recurrences, and 80 metastatic cases included in the study (Table 1), and the percentage of tumor cells ranged from 20–100%. The largest portion of the cohort consisted of lung carcinomas (35.8%) including 26 adenocarcinomas, 8 squamous cell carcinomas (SCCs), 6 small cell carcinomas, and 3 poorly differentiated carcinomas, which adequately reflects the frequency of thoracic specimens sent to our university's cytology service. The remaining cases consisted of 6 carcinomas of unknown origin, 9 high‐grade serous carcinoma (HGSC) of the ovary and fallopian tube, 1 low‐grade serous carcinoma (LGSC), 8 endometrial carcinomas (ECs) (including endometrioid, serous, and clear cell subtypes), 3 cervical carcinoma (1 squamous and 2 adenocarcinoma), 7 breast adenocarcinoma, 9 pancreatic adenocarcinoma, 6 gastrointestinal stromal tumors (GIST), 2 cholangiocarcinoma, 2 gastric adenocarcinoma, 1 low‐grade appendiceal mucinous tumor, 7 head and neck SCCs (including larynx, pharynx, ocular, and oral cavity), 2 thyroid carcinoma, 1 salivary gland neoplasms, 2 melanoma, 2 renal cell carcinoma, 1 mesothelioma, 1 urothelial carcinoma, and 3 well‐differentiated neuroendocrine tumors. A breakdown of each tumor type is represented in the pie chart (Figure 2).

### 3.2 Surgical‐Cytologic correlation

**FIGURE 3:**
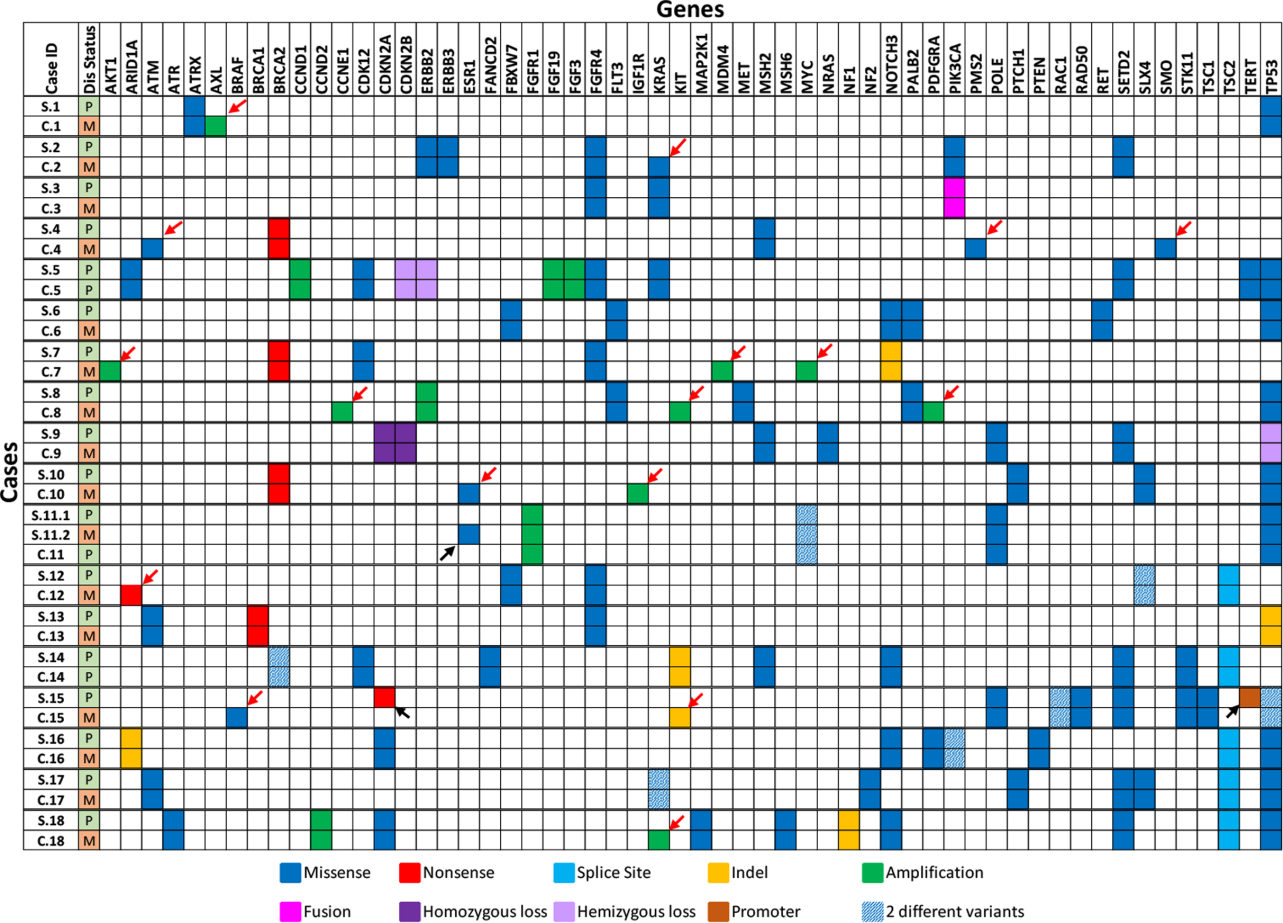
Surgical cytologic correlation. Summary of variants identified in 18 pairs of surgical and cytology samples. Each pair is separated by a double line. Disease status, primary or metastatic is indicated in the second column. Case 11 had two surgical (one primary and one metastasis) and one cytology sample sequenced. Case 5 and 14 had both samples from primary tumors, show 100% concordance. Discrepant variants are shown in black arrows (surgical) and red arrows (cytology). Each row represents a sample, columns represent genes.

There were 18 cases within the cohort that had paired surgical and cytology samples. These samples were from different time points over the course of patient treatment (ie: surgical case at primary diagnosis and cytology case at the time of metastasis), and this difference in time was noted in our correlation results table as 1st diagnosis and 2nd diagnosis (Table 2). The Spearman correlation between 1st and 2nd diagnosis was 0.65056 with a *p*‐value of <0.0001, and the Pearson correlation between 1st and 2nd diagnosis was 0.54763 with a *p*‐value of <0.0001. Both methods show a significant correlation between the surgical‐cytologic paired samples. A detailed comparison of variants detected in each of the surgical and cytology cases is shown in Figure 3. Ten out of 18 cases had discrepancy in variants between the surgical and cytology samples. In 8 cases, the cytology samples had extra variants compared to surgical samples. All 8 samples were from metastatic disease with additional copy number, missense, or nonsense variants that are likely associated with tumor progression and/or metastasis. In case 15, the surgical sample had a nonsense mutation (R58*) in CDKN2A (VAF 74.9%) and a promoter mutation (c.‐146C > T) in TERT (VAF 50.7%) that were not present in the cytology sample. The cytology sample had an indel (W557_K558del) in KIT (VAF 11.3%) and a BRAF V600E (VAF 5.3%) mutation that were not detected in the surgical sample (Figure 3). It is possible that the cytology sample represents a different clone that expanded in the tumor metastasis. Finally, for case 11, there were two surgical samples and one cytology sample. The first surgical sample and cytology samples were both from the primary breast cancer, whereas the second surgical sample was from the metastasis. In this case, the second surgical sample had a pathogenic ESR1 D538G mutation known to be associated with endocrine therapy resistance in breast cancer.^8^ The rest of the variants were identical in both surgical and cytology samples (Figure 3).

**TABLE 2:** List of variants detected in surgical and cytology paired samples

Case #	Variants at 1st diagnosis	Variants at 2nd diagnosis
1	*TP53* p.Y220C; *ATRX* p.N1860S	*TP53* p.Y220C; *ATRX* p.N1860S; ***AXL* amplification**
2	*PIK3CA* p.E542K; *ERBB3* p.V104L; *ERBB2* p.R678Q; *SETD2* p. P1962L; *SETD2* p.M1080I; *FGFR4* p.G388R	*PIK3CA* p.E542K; *ERBB3* p.V104L; *ERBB2* p.R678Q; *SETD2* p. P1962L; *SETD2* p.M1080I; *FGFR4* p.G388R; ***KRAS* p.G12V**
3	*KRAS* p.G12V; *FGFR4* p. G388R; *FNDC3B*‐*PIK3CA* fusion	*KRAS* p.G12V; *FGFR4* p. G388R; *FNDC3B*‐*PIK3CA* fusion
4	*MET* p. T1010I; *BRCA2* p. L3326*	*MET* p. T1010I; *BRCA2* p. L3326*; ***PMS2* p.H479Q; *SMO* p.V270I; *ATM* p.L1420F**
5	*KRAS* p.G12V; *TP53* p.L132R; *ARID1A* p.P120S; *SETD2* p.P1962L; *TERT* p.A279T; *FGFR4* p.G388R; *CDK12* p.L1189Q; *CCND1* Amplification; *FGF19* amplification; *FGF3* amplification; *CDKN2A* hemizygous loss; *CDKN2B* hemizygous loss	*KRAS* p.G12V; *TP53* p.L132R; *ARID1A* p.P120S; *SETD2* p.P1962L; *TERT* p.A279T; *FGFR4* p.G388R; *CDK12* p.L1189Q; *CCND1* Amplification; *FGF19* amplification; *FGF3* amplification; *CDKN2A* hemizygous loss; *CDKN2B* hemizygous loss
6	*FBXW7* p.R465Ll *TP53* p.G244S; *RET* p.E867D; *FLT3* p.I417L; *PALB2* p.P210L; *NOTCH3* p.A1020P	*FBXW7* p.R465Ll *TP53* p.G244S; *RET* p.E867D; *FLT3* p.I417L; *PALB2* p.P210L; *NOTCH3* p.A1020P
7	*BRCA2* p.R2318*; *NOTCH3* p.Y1106fs; *FGFR4* p.G388R; *CDK12* p.I1131V	*BRCA2* p.R2318*; *NOTCH3* p.Y1106fs; *FGFR4* p.G388R; *CDK12* p.I1131V; ***MDM4* amplification, *MYC* amplification, *AKT1* amplification**
8	*TP53* p.G244V; *MET* p.I316M; *FLT3* p.A988P; *PALB2* p.P210L; *NOTCH3* p.A1020P; *ERBB2* amplification	*TP53* p.G244V; *MET* p.I316M; *FLT3* p.A988P; *PALB2* p.P210L; *NOTCH3* p.A1020P; *ERBB2* amplification; ***PDGFRA* amplification; *KIT* amplification; *CNNE1* amplification**
9	*NRAS* p.Q61R; *MSH2* p.L449N; *SETD2* p.P1962L; *POLE* p.R1556W; *CDKN2A* homozygous loss; *CDKN2B* homozygous loss; *TP53* hemizygous loss	*NRAS* p.Q61R; *MSH2* p.L449N; *SETD2* p.P1962L; *POLE* p.R1556W; *CDKN2A* homozygous loss; *CDKN2B* homozygous loss; *TP53* hemizygous loss
10	*BRCA2* p.L3326*; *TP53* p.R282W; *PTCH1* p.T728M; *SLX4* p.R1761C	*BRCA2* p.L3326*; *TP53* p.R282W; *PTCH1* p.T728M; *SLX4* p.R1761C; ***ESR1* p.D538G; *IGF1R* amplification**
11	**1st Surgery:** *FGFR1* amplification, *MYC* amplification, *MYC* p.V185I, *POLE* P697S, *TP53* splice site; **2nd Surgery: *ESR1* p.D538G**, FGFR1 amplification, MYC amplification, MYC p.V185I, POLE P697S, TP53 splice site	*FGFR1* amplification, *MYC* amplification, *MYC* p.V185I, *POLE* P697S, *TP53* splice site
12	*FBXW7* p.R479P; *FGFR4* p.G388R; *SLX4* p.T919I; *SLX4* p.W546C; *TSC2* splice site	*ARID1A* p.E1718*; *FBXW7* p.R479P; *FGFR4* p.G388R; *SLX4* p.T919I; *SLX4* p.W546C; *TSC2* splice site
13	*TP53* p.R248fs; *BRCA1* p.Q1806*; *FGFR4* p.G388R; *ATM* p.B122T	*TP53* p.R248fs; *BRCA1* p.Q1806*; *FGFR4* p.G388R; *ATM* p.B122T
14	*MSH2* p.I770V; *FAND2* p.Q65H; *SETD2* p.P1962L; *BRCA2* p.H1561N; *BRCA2* p.V2138F; *CDK12* p.T1195M; *STK11* p.F354L; *NOTCH3* p.A1020P; *TSC2* splice site; *KIT* deletion	*MSH2* p.I770V; *FAND2* p.Q65H; *SETD2* p.P1962L; *BRCA2* p.H1561N; *BRCA2* p.V2138F; *CDK12* p.T1195M; *STK11* p.F354L; *NOTCH3* p.A1020P; *TSC2* splice site; *KIT* deletion
15	*RAC1* p.P29F; *RAC1* p.P29L; *CDKN2A* p.R58*; *TP53* p.E286K; *TP53* p.A159V; *SETD2* p.P1962L; *RAD50* p.D767N; *TSC1* p.K587R; *POLE* p.G6R; *STK11* p.D350N; ***TERT* promoter**	*RAC1* p.P29F; *RAC1* p.P29L; *TP53* p.E286K; *TP53* p.A159V; *SETD2* p.P1962L; *RAD50* p.D767N; *TSC1* p.K587R; *POLE* p.G6R; *STK11* p.D350N; ***BRAF* p. V600E; *KIT* p.W557_K558del**
16	*ARID1A* p.V1817fs; *PIK3CA* p.E81K; *PIK3CA* p.R88Q; *PTEN* p.Y68H; *TP53* p.R273H; *CDKN2A* p.H123Q; *PALB2* p.T386A; *NOTCH3* p.A1020P; *TSC2* splice site	*ARID1A* p.V1817fs; *PIK3CA* p.E81K; *PIK3CA* p.R88Q; *PTEN* p.Y68H; *TP53* p.R273H; *CDKN2A* p.H123Q; *PALB2* p.T386A; *NOTCH3* p.A1020P; *TSC2* splice site
17	*KRAS* p.G12F; *KRAS* p.G12C; *TP53* p.R273L; *SETD2* p.P1962L; *PTCH1* p.T728M; *ATM* p.M1040V; *SLX4* p.P975L; *NF2* p.E463L; *TSC2* In/Del	*KRAS* p.G12F; *KRAS* p.G12C; *TP53* p.R273L; *SETD2* p.P1962L; *PTCH1* p.T728M; *ATM* p.M1040V; *SLX4* p.P975L; *NF2* p.E463L; *TSC2* In/Del
18	*CDKN2A* p.D108H; *MAP2K1* p.P124L; *TP53* p.L194R; *NF1* p.Y80fs; *MSH6* p.S63P; *SETD2* p.P1962L; *ATR* p.S1607N; *NOTCH3* p.A1020P; *TSC2* splice site; *CCND2* amplification	*CDKN2A* p.D108H; *MAP2K1* p.P124L; *TP53* p.L194R; *NF1* p.Y80fs; *MSH6* p.S63P; *SETD2* p.P1962L; *ATR* p.S1607N; *NOTCH3* p.A1020P; *TSC2* splice site; *CCND2* amplification; ***KRAS* amplification**

*Note*: Bolded text identifies variants not identified in both specimens.

### 3.3 Cytology cases

**FIGURE 4:**
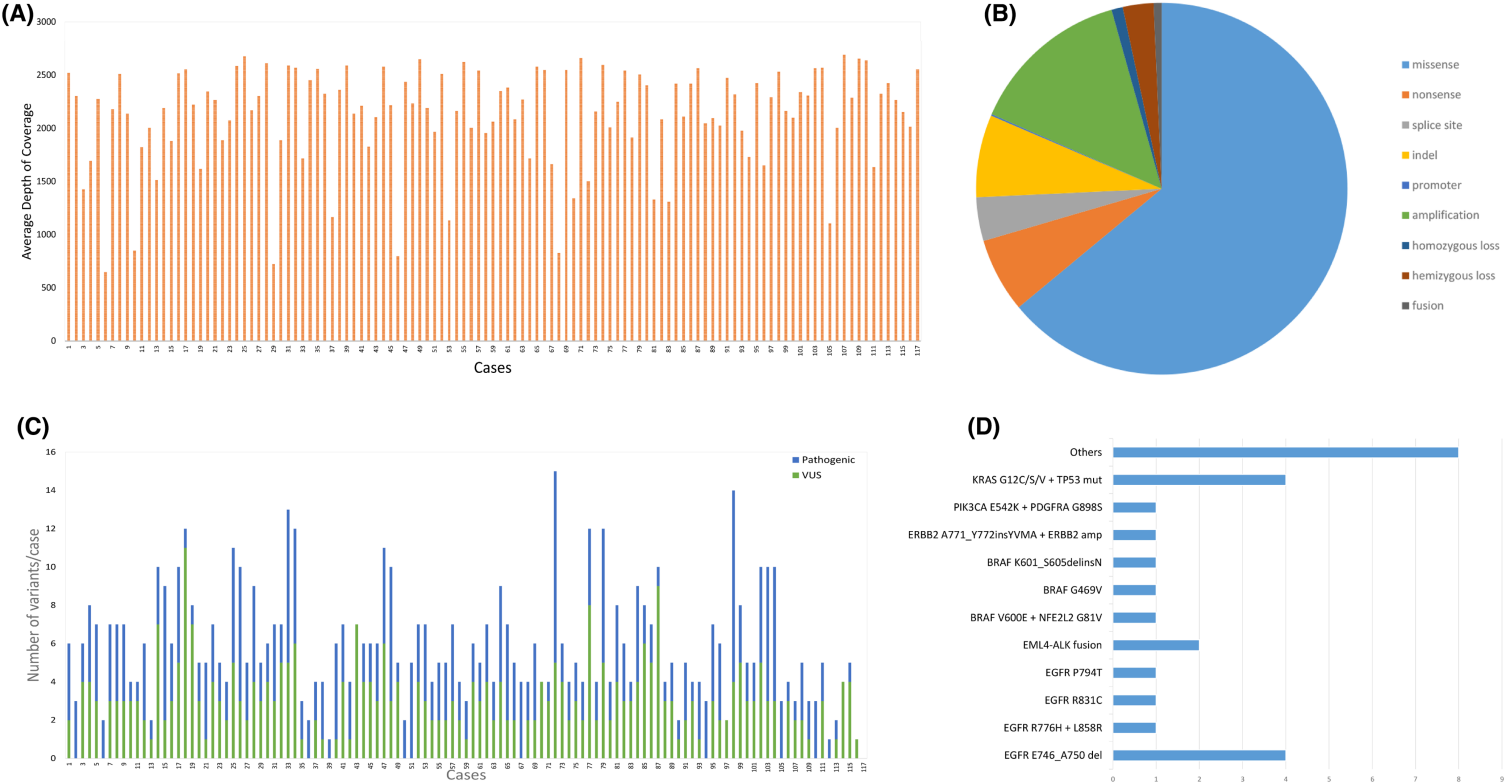
Performance metrics and variants detected in the case cohort. (A) Histogram showing average depth of coverage in all samples (range 648x–2694x). Cases are grouped by diagnosis and are in the same order as shown in Figure 1; X‐axis: cases 1–117; Y‐axis: Depth of coverage; (B) Pie chart showing the types of variants detected in the cohort; (C) Histogram showing the number of pathogenic variants and VUS for each case as well as the total number of variants identified for each case. Each case has a single bar representing the total number of variants and color difference within the bar indicates the types of variants present; (D) Clinically actionable variants detected in the lung adenocarcinoma.

**FIGURE 5:**
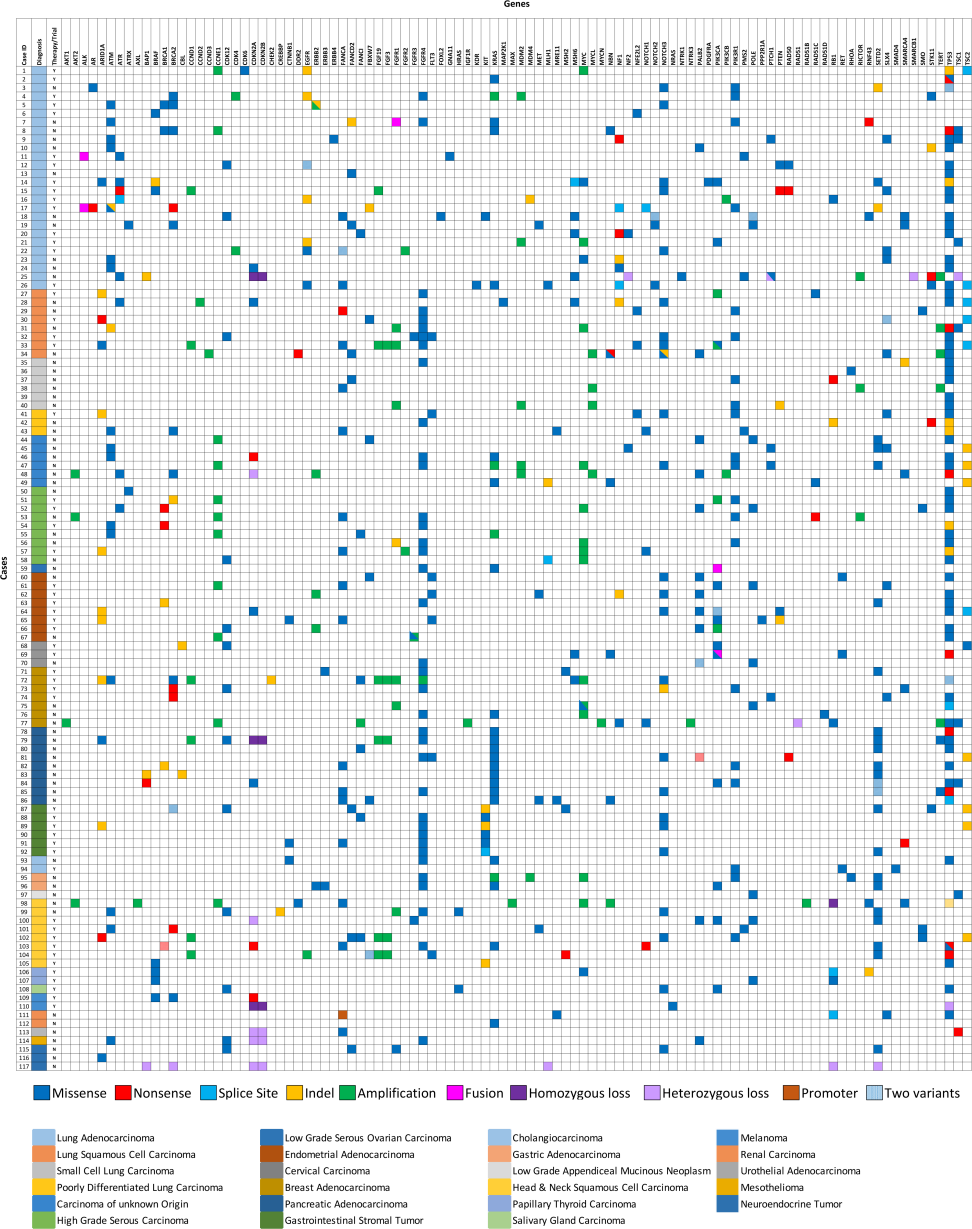
Heatmap of all variants detected in the cohort of 117 cases. Cases are grouped by diagnosis and are shown in the same order as in Figure 1. Each row represents a case, columns represent genes. Second column represents diagnosis and third column shows clinical relevance based on the presence of variants. Multiple variants of the same gene are indicated by stripes if the two variants are of the same type or by triangles if they are of different types.

Within the 117 cases sequenced, the depth of coverage ranged from 648X to 2694X (Figure 4A). A total of 711 variants were detected, including 505 single nucleotide variants (SNVs), 2 multinucleotide variants (MNVs), 73 insertion/deletions (indels), 126 copy number variants (CNVs), and 5 fusions (Figure 4B, Figure 5, Table S1). There was a total of 347 pathogenic variants and 345 variants of undetermined significance (VUS) detected in all cases (Figure 4C). The 20 negative control samples were sequenced with adequate depth of coverage and no pathogenic variants were detected.

In the entire cohort of 117 cases, TP53 was the most frequently mutated (58%) gene (Figure 5). Among the 26 lung adenocarcinomas, 18 cases had clinically actionable variants for which FDA‐approved drugs are available. Seven cases had *EGFR* mutations (4 cases with exon 19 deletion, 1 case with R776H and L858R, 1 case with R831C, and 1 case with P794T). Four cases with *KRAS* G12C/S/V and TP53 variants, 2 cases with *EML4‐ALK* fusion, 3 *cases with BRAF* variants (1 V600E, 1 G469V, and 1 exon 15 indel), 1 case with *PIK3CA* E542K, and 1 case with *ERBB2* exon 20 indel. In the remaining 8 cases variants in *NF1*, *STK11*, *ERBB4*, *SETD2*, *BAP1*, and *CDKN2A* genes were detected (Figure 4D, Table S1). One case with *KRAS* G12S also had a *WHSC1L1‐FGFR1* fusion. The squamous cell lung carcinomas showed two cases with *ARID1A* loss of function variants (E1718* and G1848fs), one *FGFR3* S249C variant, one *MAP2K1* P124L, and one case with *PIK3CA* amplification and E545A variant. One case of poorly differentiated carcinoma of the lung had an *ARID1A* G836fs variant. The small cell carcinoma cases and remaining lung carcinoma cases did not have clinically relevant pathogenic variants.^9^

Of the 6 carcinomas of unknown origin, 2 cases showed a *KRAS* G12D/V variant and one case showed an amplification of the same gene. One case had an *ERBB2* amplification with a *SMARCA4* loss of function G1232C variant. One case had many amplifications present.

Our cohort of gynecologic tumors included 9 high‐grade serous ovarian/fallopian tube cancers (HGSC), one LGSC, 8 ECs, one SCC of the cervix, and two adenocarcinomas of the cervix. Of the HGSC, two cases had a *BRCA1* pathogenic variant (W1733* and Q1806*), and one case had a *BRCA2* pathogenic variant (L1227fs). Additionally, one case showed a *FGFR2* amplification and another case showed a *FGFR1* pathogenic variant (D166del). The LGSC showed a *PIK3CA*‐*FNDC3B* fusion and a *KRAS* G12V variant. Of the 8 ECs, two had the most common hotspot *PIK3CA* variants (Q546K and E542A), one case with two different hotspot *PIK3CA* variants (E81K and R88Q) and an additional case with a *PIK3CA* amplification. One case of EC had a *BRCA1* frameshift variant (E1210fs), and one case showed *ERBB2* amplification. *FGFR3* amplification and gain‐of‐function variant in one case appeared to be the driving alterations. Within the 7 breast cancer cases, two cases showed a *BRCA2* nonsense variant (p.R2318* and p.K3326*), one case showed ERBB3 G234R, one case showed KRAS G12V, and one case showed ARID1A P453fs. No *PIK3CA*, *BRCA1*, or *ERBB2* alterations were identified in the breast carcinoma cases.^10^, ^11^ Amplifications of *AKT1, MYCN, MYC, FGFR1, FGFR4, FGF3, FGF19, IGF1R, CCND1, CCNE1, TERT*, and *NTRK3* were detected in 4/7 cases (Figure 5).

All nine pancreatic adenocarcinomas harbored exon 2 pathogenic gain‐of‐function variants in *KRAS* and 6/9 cases had a deleterious loss‐of‐function variant in *TP53*. In addition to these variants, one case showed a frameshift mutation in *BRCA1* (Q867fs), and another case showed a less common G1049R gain‐of‐function variant in *PIK3CA*. All six cases of GIST harbored a pathogenic variant in *KIT*—three indels including a splice site variant and 3 gain of function missense variants. Of the two cholangiocarcinoma cases, one case had a *SMAD4* R361H pathogenic variant and the other case had a pathogenic *KRAS*, *TP53*, and additional *CTNNB1* S45F variant. The two gastric carcinoma cases showed an *ERBB2* R678Q pathogenic variant with a *PIK3CA* E542K and *KRAS* G12V variants.

Within the SCCs of the head and neck region, there was variability in locations that was reflected in the variants detected. One case from the face showed two loss of function variants in *BRCA1* (E17758* and E1491*). A frontal sinus‐based neoplasm had a loss of function *BRCA2* K3326* variant. A nasopharyngeal‐based lesion harbored a pathogenic *HRAS* Q61R variant and a *FGFR1* amplification. A laryngeal SCC was found to have a *PIK3CA* H1047R pathogenic variant. The additional larynx‐based tumor and the floor of mouth tumor included in our cohort showed significant number of copy number variations (CNVs).^12^

In the smaller diagnosis cohort, one melanoma case had a *NRAS* Q61R variant and the other case of melanoma had a *BRAF* V600E variant. Both papillary thyroid carcinoma cases harbored *BRAF* V600E variants. One case of renal cell carcinoma harbored a *KRAS* G12D variant and showed an oncocytic papillary phenotype. The salivary gland malignancy showed *PIK3CA* H1047R and *HRAS* Q61R pathogenic variants.

Of note, there were no significant pathogenic variants detected in the urothelial carcinoma, the mesothelioma, the low‐grade appendiceal mucinous tumor, and neuroendocrine tumors. A summary of variants along with diagnosis and clinical relevance is shown in Figure 5, and for a full list of variants detected in our cohort, please refer to Table S1.

## 4 DISCUSSION

In recent years, cytology samples are a growing proportion of samples that are sent for routine molecular diagnostics. Our study demonstrates that NGS‐based targeted sequencing can be performed in a highly accurate and reproducible manner with small quantity cytology samples. Within our university cytology service, we found that approximately 27% (120/451) of our malignant cytologic samples had cell block material adequate for molecular testing via a NGS comprehensive assay. Of the 117 cases included in this study, 18 cases had a surgical pathology sample available and showed good concordance of results between cytologic and surgical pathology specimens validating the utility of cytologic specimens in the molecular diagnostics laboratory.

One of the challenges of using comprehensive NGS‐based assays for routine diagnostic testing is the quality and quantity of tissue required for detecting clinically relevant mutations. Our study demonstrates that we had a very small failure rate (2.5%; 3/120) due to either sample size or formalin‐induced artifacts. Our assay detected variants (SNVs) with 5% or higher allele frequency, CNVs (amplification, single copy loss and homozygous loss) and gene fusions efficiently. For many tumors, the percent tumor cells present in the section amounted to 20% but we were able to enrich the tumor fraction by macrodissecting to yield sufficient DNA and RNA for running the assay successfully.

Genomic heterogeneity plays a significant role in eventual drug resistance and treatment failure resulting from the generation of subpopulations within a tumor. The Cancer Genome Atlas (TCGA) studies performed by inter‐ and intra‐tumor comparisons have shown tumor heterogeneity in many types of tumors including lung, breast, prostate, glioblastoma and colon cancers.^10^, ^13^, ^14^, ^15^, ^16^ Furthermore, TCGA pan‐cancer analysis of genomic landscapes of 12 tumor types from more than 3000 tumors identified 127 significantly mutated genes with both established and emerging links to cancer, indicating that the number of driver mutations required for oncogenesis is relatively small.^17^ Although a common set of driver mutations exists in a given cancer type, the combination of mutations within a patient tumor and their distribution within the founding clone and subclones will be critical for optimizing their treatment. We sequenced the samples at a higher depth of coverage to account for the sub‐populations of cells that might be contributing toward the makeup of the tumor.

One of the limitations with cytology samples is the small quantity of cellular material, which raises concerns about capturing tumor heterogeneity. In FNA biopsy samples, multiple planes of the tumor and the resulting specimen comprises multiple passes through the tumor. In body fluid specimens, concern for heterogeneity should be low because malignant cells in cavity fluids typically represent the most aggressive and metastatic subclone of the tumor and would be the ideal subpopulation to study. Methodologically, increasing the percent of tumor cells in a sample by selective microdissection proportionally increases the yield of tumor cells coming from the major subpopulations, thereby increasing the yield of both driver and passenger mutations.^18^

In our cohort, we identified 32 cases (27.4%) that would qualify for FDA‐approved targeted therapy according to the current guidelines. An additional 23 cases (19.7%) would qualify for a clinical trial based on pathogenic variants detected by NGS (Table 3). With approximately 45% of cases in our cohort qualifying for some form of targeted therapy, the critical importance of providing high complexity NGS testing of cytologic samples is undeniable. Currently available treatments include imatinib for *KIT* alterations in GIST, gefitinib, osimertinib, sotorasib, crizotinib, for alterations in EGFR, KRAS, and ALK fusion in NSCLC, PARP inhibitors, and alpelisib for *BRCA1/2* and *PIK3CA* variants in gynecologic malignancies, and dabrafenib and trametinib for *BRAF* V600E in melanoma and metastatic papillary thyroid carcinoma.^19^, ^20^, ^21^, ^22^, ^23^, ^24^Our cohort does not include enough samples for each diagnosis to reach statistical significance with regard to clinical outcome. But comparing cases with and without actionable variants in our overall cohort, there was a significant correlation between cases with targetable variants with both PFS and OS (Figure S1).

**TABLE 3:** Cases with variants for which targeted therapy or clinical trial available

Case ID	Diagnosis	Clinical/Pathologic Stage at Diagnosis	Primary/Metastasis	Gene	Exon	Variant type	AA Change	FDA‐Approved Therapy Options	Clinical Trial Options
1	Lung Adeno	Stage IVB; pT4pM1c	Met	EGFR	19	INDEL	p.E746_A750del	Yes	
2	Lung Adeno	pT2apN0	Recurrence	KRAS	2	SNV	p.G12C	Yes	
4	Lung Adeno	Stage IVB	Met	EGFR	19	INDEL	p.E746_A750del	Yes	
5	Lung Adeno	Stage IV	Met	ERBB2	20	INDEL	p.A771_Y772insYVMA	Yes	
6	Lung Adeno	Stage IIIA	Met	BRAF	15	SNV	p.V600E	Yes	
11	Lung Adeno	Stage IVB	Met	EML4‐ALK	e13|e20	FUSION	Read count: 27218	Yes	
12	Lung Adeno	Stage IV	Primary	EGFR	21	SNV	p.L858R	Yes	
14	Lung Adeno	Stage IVA	Primary	PIK3CA	10	SNV	p.E542K		Yes
15	Lung Adeno	Stage IV	Met	BRAF	11	SNV	p.G469V	Yes	
16	Lung Adeno	Unknown	Primary	EGFR	19	INDEL	p.E746_A750del	Yes	
17	Lung Adeno	Stage IVB; pT2b pN3 pM1c	Primary	EML4‐ALK	e13|e20	FUSION	Read count: 131144	Yes	
20	Lung Adeno	Stage I, pT1 pN0	Recurrence	NF1	37	INDEL	p.C1682Ter		Yes
21	Lung Adeno	Stage IVB	Met	EGFR	19	INDEL	p.E746_A750del		Yes
22	Lung Adeno	At least Stage III	Met	EGFR	21	SNV	p.R831C	Yes	
23	Lung Adeno	Stage IIIA	Primary	NF1	42	INDEL	p.L2103fs		Yes
26	Lung Adeno	Stage IV	Met	KRAS	2	SNV	p.G12C	Yes	
27	Lung SqCC	Stage IIIB	Recurrence	ARID1A	20	SNV	p.G1848fs		Yes
30	Lung SqCC	Stage IIIB	Met	ARID1A	20	SNV	p.E1718*		Yes
32	Lung SqCC	Stage IIIA	Primary	FGFR3	7	SNV	p.S249C		Yes
33	Lung SqCC	At least Stage IIIA	Primary	PIK3CA	10	SNV	p.E545K		Yes
41	Poorly differentiated lung ca	Stage IV	Met	ARID1A	8	SNV	p.G836fs		Yes
51	High‐grade serous ovarian ca	Stage IVA	Met	BRCA2	11	SNV	p.L1227fs	Yes	
52	High‐grade serous ovarian ca	Stage IV	Met	BRCA1	18	SNV	p.W1733*	Yes	
54	High‐grade serous ovarian ca	Stage IV	Met	BRCA1	22	SNV	p.Q1806Ter	Yes	
57	High grade serous ovarian ca	Stage IIIC	Met	ARID1A	1	SNV	p.A246fs		Yes
61	Endometrial adenocarcinoma	Stage IV	Met	PIK3CA	10	SNV	p.E542A	Yes	
62	Endometrial adenocarcinoma	Stage IV	Met	ERBB2	Amp	CNV	Copy number: 16	Yes	
63	Endometrial adenocarcinoma	Stage IV	Met	BRCA1	10	SNV	p.E1210fs	Yes	
64	Endometrial adenocarcinoma	Stage IV	Met	ARID1A	20	SNV	p.V1817fs		Yes
65	Endometrial adenocarcinoma	Stage IV	Met	PIK3CA	10	SNV	p.Q546K	Yes	
66	Endometrial adenocarcinoma	Stage IVB, pT3pM1	Met	ERBB2	Amp	CNV	Copy number: 8.4	Yes	
68	Cervical SqCC	Unknown	Met	PIK3CA	10	SNV	p.E545K		Yes
69	Cervical SqCC	Stage IV	Met	PIK3CA	10	SNV	p.E542V		Yes
71	Breast adenocarcinoma	Stage IV	Met	ERBB3	7	SNV	p.G284R		Yes
72	Breast adenocarcinoma	Unknown	Met	ARID1A	3	SNV	p.P453fs		Yes
73	Bilateral breast adenocarcinoma	pT1c	Met	BRCA2	13	SNV	p.R2318*	Yes	
74	Breast adenocarcinoma	Stage IIB	Met	BRCA2	27	SNV	p.K3326*	Yes	
87	Gastro intestinal stromal tumor	pT2pN0	Primary	KIT	11	INDEL	p.W557_K558del	Yes	
88	Gastro intestinal stromal tumor	Unknown	Primary	KIT	11	SNV	p.V559G	Yes	
89	Gastro intestinal stromal tumor	Stage IV	Primary	KIT	11	INDEL	p.W557_K558del	Yes	
90	Gastro intestinal stromal tumor	Unknown	Primary	KIT	11	SNV	p.W557G	Yes	
91	Gastro intestinal stromal tumor	pT2	Primary	KIT	11	SNV	p.V559G	Yes	
92	Gastro intestinal stromal tumor	Unknown	Recurrence	KIT	11	INDEL	Splice site	Yes	
94	Cholangiocarcinoma	Stage IV	Met	RET	13	SNV	p.Y791F		Yes
100	Laryngeal SCC	pT4apN0	Met	PIK3CA	21	SNV	p.H1047R		Yes
101	Left frontal sinus SCC	pT4apN0pM0	Met	MET	14	SNV	p.T1010I		Yes
102	Tongue/tonsil/FOM SCC	Stage IVB	Met	ARID1A	9	SNV	p.Q944*		Yes
103	SCC of face (skin)	pT2pN0	Met	BRCA1	20	SNV	p.E1775*		Yes
104	SCC of larynx	Stage IVA; pT2pN2b	Met	MSH2	15	SNV	p.C873*		Yes
105	Conjunctival SCC	pT4pN1	Met	KIT	11	INDEL	p.W557_K558del		Yes
106	Metastatic papillary thyroid ca	Stage III, pT4 pN1a	Recurrence	BRAF	15	SNV	p.V600E	Yes	
107	Papillary thyroid ca	Unknown	Met	BRAF	15	SNV	p.V600E	Yes	
108	High‐grade salivary ca	At least Stage III	Met	PIK3CA	21	SNV	p.H1047R		Yes
109	Melanoma	Unknown	Met	BRAF	15	SNV	p.V600E	Yes	
110	Melanoma	Unknown	Met	NRAS	3	SNV	p.Q61R	Yes	

It is important to note that 50% of the cases that came through the cytology service during the period of archival review were not included in this study due to lack of material to review or due to insufficient tissue for further analysis. Because of the retrospective nature of this study, some samples had cell blocks that were exhausted or had very little tissue left that was inadequate for molecular testing. Additional prospective studies would need to be done to get a more accurate success rate of cytologic cell blocks in light of tissue preservation methods used in laboratories today to improve ancillary testing results. Alternative forms of cell preservation and additional FNA passes dedicated for molecular laboratories could also increase the number of cytologic cases for successful molecular analysis. At our institution, a high number of patients diagnosed by the cytology service as “positive for malignancy” are high stage at presentation or have recurrent disease, and these patients would benefit most from molecular analysis. Although intra‐tumor heterogeneity is a concern, molecular analysis of cytologic specimens can adequately identify driver mutations for which targeted therapy options are available. As shown in our comparison of surgical samples and cytologic cell blocks, the major drivers of oncogenesis in each tumor pair were identified in both samples. As most of the patients in our cohort either were not eligible for surgical resection or had not responded to first‐line chemotherapy at the time of the cytologic sample collection, this is exactly the cohort that would most benefit from approved targeted therapies or clinical trial options. With this in mind, and extrapolating from our study, approximately 30% more of the patients in our population with similar clinical histories would qualify for currently approved targeted therapy regimens or would qualify for a clinical trial. We continue to optimize our service for such patients and have developed confidence in the methods and yield of targeted sequencing of solid tumor in FNA and body fluid cytology cell block specimens.

## AUTHOR CONTRIBUTIONS

**Erica Vormittag‐Nocito:** Conceptualization (supporting); methodology (supporting); writing – original draft (supporting); writing – review and editing (supporting). **Ravindra Kumar:** Data curation (supporting). **Kunwar Narayan:** Investigation (supporting); project administration (supporting). **Zhengjia Chen:** Software (lead). **Odile David:** Project administration (supporting); resources (supporting); supervision (supporting). **Frederick Behm:** Resources (lead); supervision (supporting). **Gayatry Mohapatra:** Conceptualization (equal); data curation (equal); formal analysis (equal); project administration (equal); writing – original draft (lead); writing – review and editing (lead).

## CONFLICT OF INTEREST

None.

## ETHICAL APPROVAL STATEMENT

This study was approved by the University of Illinois Institutional Review Board with an exemption granted for written informed consent.

## CLINICAL TRIAL REGISTRATION

N/A

## Supporting information


Figure S1

Table S1

## Data Availability

Data will be shared once the manuscript is accepted.
