# Supplementary figures and images for: Utilization of cytologic cell blocks for targeted sequencing of solid tumors
Source: Cancer Med. 2022 Sep 20;12(4):4042–63. doi: 10.1002/cam4.5261 (PMC9972011; doi:10.1002/cam4.5261)

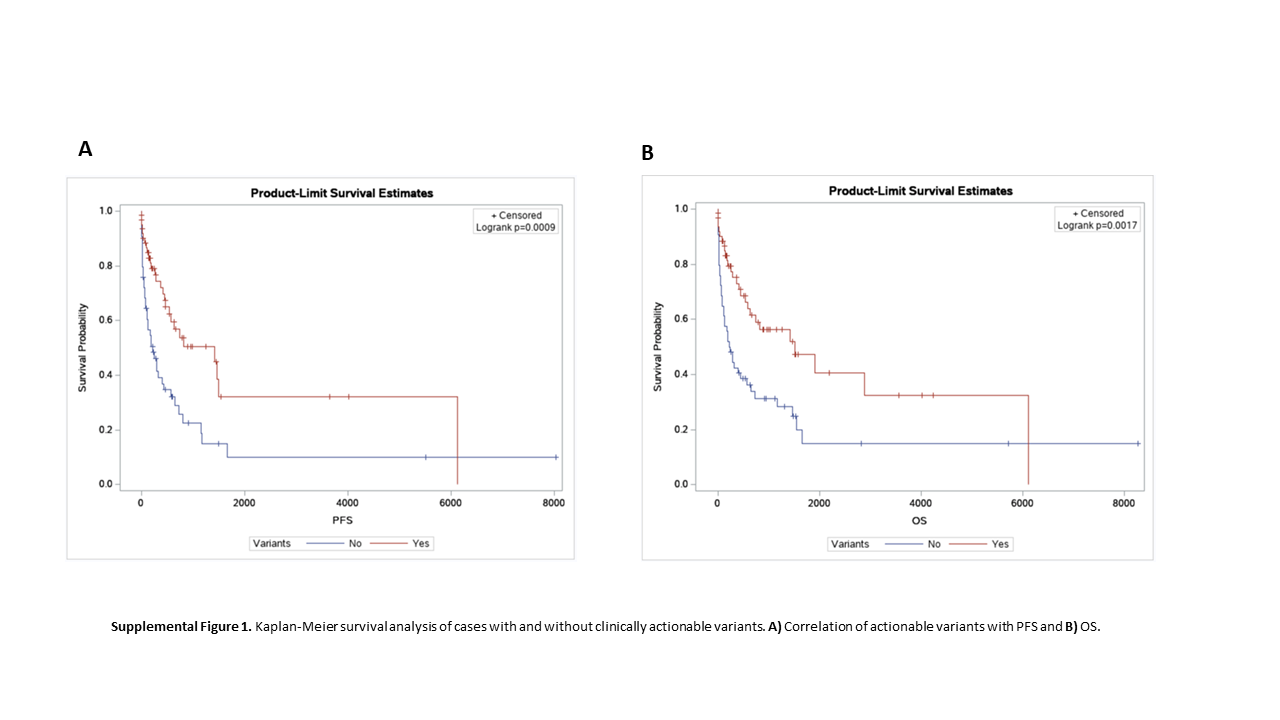

Supplement: Supplementary file 1 — Figure S1 [file CAM4-12-4042-s002.png]
